# A Scoping Review of Community-Based Adult Suicide Prevention Initiatives in Rural and Regional Australia

**DOI:** 10.3390/ijerph19127007

**Published:** 2022-06-08

**Authors:** Elissa Dabkowski, Joanne E. Porter, Michael S. Barbagallo, Valerie Prokopiv, Megan R. Jackson

**Affiliations:** Collaborative Evaluation and Research Group, Gippsland Campus, Federation University Australia, Churchill 3842, Australia; joanne.porter@federation.edu.au (J.E.P.); m.barbagallo@federation.edu.au (M.S.B.); v.prokopiv@federation.edu.au (V.P.); m.jackson@federation.edu.au (M.R.J.)

**Keywords:** suicide, prevention, community-based, rural, regional, scoping review

## Abstract

The need for continued research into suicide prevention strategies is undeniable, with high global statistics demonstrating the urgency of this public health issue. In Australia, approximately 3000 people end their lives each year, with those living in rural and regional areas identified as having a higher risk of dying by suicide. Due to decreased access and support services in these areas, community-based suicide prevention initiatives provide opportunities to educate and support local communities. A scoping review was conducted to explore the literature pertaining to such programs in rural and/or regional communities in Australia. This review follows the five-stage Arksey and O’Malley (2005) framework and the Preferred Reporting Items for Systematic Reviews and Meta-Analyses extension for Scoping Reviews (PRISMA-ScR) checklist. Nine databases were searched, from which studies were considered eligible if suicide prevention programs were community-based and catered for adults (aged ≥ 18 years) in rural or regional Australia. Ten papers that met our inclusion criteria were included in this review, showcasing a variety of interventions such as workshops, a digital intervention, art therapy, and initiatives to increase education and reduce stigma around suicide. Program engagement strategies included the importance of providing culturally appropriate services, the inclusion of lived experience mentoring, and tailoring the suicide prevention program to reach its targeted audience. Overall, there is a dearth of literature surrounding community-based suicide prevention initiatives for adults in rural and regional Australia. Further evaluation of community-based projects is required to ensure quality improvement and tailored suicide prevention initiatives for rural and regional Australians.

## 1. Introduction

In Australia, suicide is identified as the leading cause of death for people aged between 15 and 49 years [1]. Approximately 3000 Australians end their lives each year—an average of eight deaths per day [1]. Suicide was also one of the five leading causes of death for Indigenous Australians between 2012 and 2016 [2]. These statistics are reflected globally, with the World Health Organization (WHO) reporting 703,000 deaths by suicide each year [3]. The WHO estimates that a person dies by suicide every 40 s, with an additional 20 non-fatal suicide attempts for every reported suicide [4]. It is important to note that these statistics are likely to be underreported because of stigma and insufficient surveillance and monitoring systems [3]. These statistics indicate the severity of this public health issue and the need to prioritise suicide prevention efforts. The effects of suicide are far-reaching, and have long-lasting effects on families and often entire communities [3].

High-income countries have the highest rate of suicide, at 11.5 deaths per 100,000 people, with males three times as likely to die by suicide compared to females [4]. In addition, people living in rural and regional areas experience poorer mental health and wellbeing due to challenges in accessing treatment and support [5]. A Royal Commission was established in 2019 to investigate the mental health system in the state of Victoria, due to concerns of insufficient support for people living with mental illness, their families, and mental health workers [5]. This Royal Commission established that suicide rates are higher in rural and regional Victoria compared to metropolitan Melbourne [5]. One of the key findings was that communities do not adequately support good mental health and wellbeing [5]. The social factors that influence mental health are not recognised, which subsequently diminishes the importance of community and workplaces to mental health [5].

Suicide prevention initiatives require a holistic and multifactorial approach. As part of *Australia’s Long Term National Health Plan*, the Australian Government has invested AUD 55 million in national suicide prevention trials, and established the National Suicide Prevention Research Fund to disseminate research pertaining to suicide prevention [6]. An Australian report into research priorities in suicide prevention recommended that funding should be allocated and prioritised for studies that evaluate interventions [7]. It is essential that suicide prevention initiatives are evaluated after their implementation for continuous quality-improvement purposes, and to optimise health outcomes [8]. Combinations of evidence-based suicide prevention strategies should be assessed at both the individual and community levels, with robust research designs [9].

There is a dearth of peer-reviewed publications on community co-produced mental health initiatives in rural Australia, despite an increased number of initiatives that target early intervention, education, and suicide prevention [10]. Given the high priority of this public health issue, this review explores the literature pertaining to suicide prevention initiatives in rural and/or regional communities in an Australian context. This differs from previous reviews such as those by De Cotta et al. [10] or Roy et al. [11], as the authors were interested in community suicide prevention initiatives that focus on rural/regional adults, the key characteristics of these programs, and the strategies used to engage their targeted populations. As such, a scoping review was the preferred typology to map these key concepts. Identifying these key characteristics and strategies could help to inform future delivery of suicide prevention initiatives for rural and regional Australians. The findings of this scoping review may help to provide recommendations for the implementation and evaluation of future suicide prevention programs, and to identify research gaps in the literature.

## 2. Method

This scoping review was conducted using the five-stage methodological framework set out by Arksey and O’Malley [12]. It was reported according to the Preferred Reporting Items for Systematic Reviews and Meta-Analyses extension for Scoping Reviews (PRISMA-ScR) checklist [13]. The sixth stage of a scoping review—stakeholder consultation—is an optional element and was not included in this review.

### 2.1. Identifying the Research Question

The research question for this scoping review was “what is known from the literature about community-based suicide prevention initiatives for adults in Australian rural and regional communities?” The specific research questions for this review were as follows: (1) What are the characteristics of suicide prevention initiatives in Australian rural and regional communities? (2) How are these suicide prevention initiatives measured to determine their overall efficacy for their targeted population?

### 2.2. Identifying Relevant Studies

This literature search was conducted between November 2021 and February 2022, with the last search conducted on 12 February 2022. A scoping review protocol was not published for this review; however, the authors collectively devised the search strategy in consultation with a research librarian. The search included six EBSCOhost databases: Academic Search Complete, CINAHL Complete, ERIC, MEDLINE, APA PsycArticles, and APA PsycInfo. Other databases searched include Scopus, Web of Science, and the search engine Google Scholar. Databases were searched using a Boolean search strategy, in which key concepts, truncations, and variations were entered into the databases (Table 1). The authors also manually searched the reference lists of included papers and other reviews to identify further eligible papers.

### 2.3. Study Selection

The inclusion and exclusion criteria were developed by the authors E.D., J.E.P., and M.S.B., and are shown in Table 2. A 10-year search period was selected to review the current evidence. A longer time period is typically utilised to map the scope of the literature; however, the authors preferred a shorter date range to establish the latest evidence on this important topic. Although scoping reviews have the propensity to include grey literature, the research team opted to include only peer-reviewed papers to improve the rigour of the review.

Non-original studies and study protocols were excluded; however, their reference lists were manually searched for potential papers. The authors were interested in community-based suicide prevention initiatives only; therefore, papers that focused on general themes or epidemiological studies were excluded. Interventions involved with primary care settings, hospitals, or outpatient settings were also excluded from this review. General suicide prevention programs that were national-based and referred to a generalised population were excluded, as the focus of this review was rural-based only. The search results were uploaded into EndNote, and duplications were removed. Two authors completed an independent title and abstract screening, where a third author moderated the process until consensus was reached. The approved screened records were obtained in full text and further evaluated by the research team to determine their relevance to the scoping review’s aims. The authors did not contact other authors of included papers to identify any additional sources. All authors approved the final list of articles for this scoping review.

### 2.4. Charting the Data

To chart the data, two extraction forms were initially developed by the authors E.D. and M.S.B. This was an iterative process, in which the data categories were revised after reviewing the included articles. The data were extracted verbatim by the lead author, and were independently verified by the author J.E.P. A quality appraisal was not conducted, as this is not always part of the scoping review typology [12]. Table 3 and Table 4 present the charted data in line with the research objectives for this review.

### 2.5. Reporting the Results

The fifth step of the scoping review framework involves collating, summarising, and reporting the results [12]. After charting the data in the two summary tables, the authors reported on the general article characteristics. A narrative synthesis was used to analyse the results under the following headings: Key Characteristics of Suicide Prevention Initiatives, Program Evaluation, Program Strategies, and Key Findings of Initiatives. A narrative synthesis adopts textual descriptions to communicate the findings of the included studies, and can be useful to discuss similarities and differences [14].

## 3. Results

**Figure 1:**
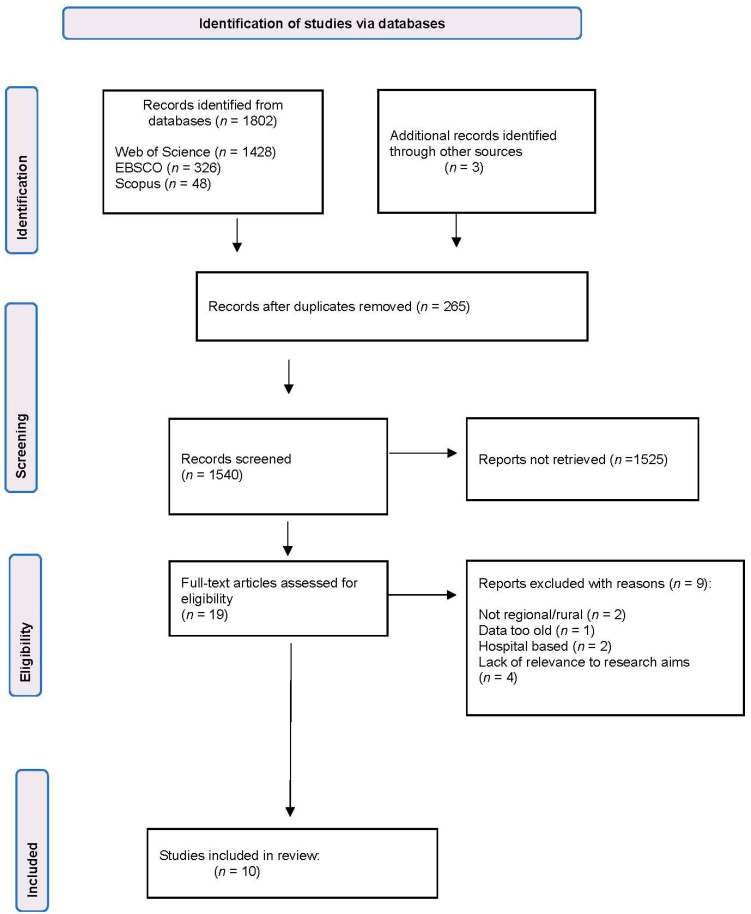
Modified PRISMA flowchart of the search strategy [15].

From the search process, 10 studies were identified that met the inclusion criteria. Figure 1 shows the PRISMA flowchart of our search strategy. Table 3 details the data summary obtained from these 10 studies, including the study aims, study design, description of suicide prevention initiative, and main findings. Table 4 maps the key characteristics of the suicide prevention programs, including the frequency of the program, targeted audience, evaluation plan/measurement/tool, program strategies for engagement, and lessons learnt. The data obtained from these studies are summarised below.

### 3.1. Article Characteristics

The 10 included studies were within a six-year time period (2016 to 2021), in which half of the studies were of a mixed-methods design (*n* = 5). Three studies were of a pre- and post-intervention evaluation design, along with one descriptive case study. A commentary describing the lessons learned from a suicide prevention program spanning a 14-year period was also included [16]. Aboriginal and Torres Strait Islander people were the intended target population in four of the suicide prevention initiatives [17,18,19,20]. One study [21] discussed how adult males in farming communities were the intended target population; however, it was decided to allow female participation to avoid potential harm from exclusion. Two studies reported on the same suicide prevention initiative [22,23]; however, it was decided to include both studies in this review due to the differing focus of the studies. This was also the case with the works of Perceval et al. [24] and Handley et al. [16].

Most suicide prevention initiatives took place in rural and regional New South Wales [16,17,18,24,25]. One initiative occurred in Echuca, Victoria [19], and the same initiative in Tasmania was evaluated twice [22,23]. The online digital intervention in Kennedy et al. [21] was designed to engage people in rural and regional farming communities. The community workshops in Snodgrass et al. [20] featured in 40 rural and remote locations in Australia.

**Table 3:** Data summary of the included studies.

Author and Year	Study Aim(s)	Study Design	Population	Description of Suicide Prevention Initiative	Main Findings
Barnett et al. [22] 2019	To gain an understanding of the experience of artists impacted by mental illness who participated in the Rural Art Roadshow.	Mixed methods	*n* = 23 artists (17.4% male)	The aim of the project was to help reduce stigma and promote a positive image of mental health in rural communities.	The three themes—Community Impact, Social Gains, and Personal Gains—demonstrated positive social and personal benefits for participating artists. The Rural Art Roadshow is a promising mental health promotion approach that could be replicated in other rural and remote areas of Australia.
Calabria et al. [17] 2020	To examine the feasibility and acceptability of the Aboriginal-adapted Community Reinforcement Approach (CRA) delivered to Aboriginal and non-Aboriginal clients in a non-Aboriginal-focused rural, community-based drug and alcohol treatment service, and to assess pre–post-program changes for drug and alcohol use and wellbeing outcomes.	Quantitative pre- and post-intervention evaluation	Aboriginal and non-Aboriginal clients aged 18 years and over (*n* = 55, 58% males)	The CRA is an evidence-based cognitive behavioural therapy (CBT) intervention that targets harmful drug and alcohol use. Compared to the US version, the tailored CRA had reduced technical language, reduced number of treatment sessions, the option of a group delivery, and was deemed to be culturally acceptable.	The CRA was associated with statistically significant reductions in the use of alcohol, tobacco, cannabis, amphetamines, and over the counter medications, as well as levels of psychological distress. The CRA was also associated with an increase in levels of empowerment for both Aboriginal and non-Aboriginal clients.
Davies et al. [18] 2020	To evaluate the We-Yarn suicide prevention gatekeeper training Workshop, and to examine whether participants reported being more able to address and respond to suicide in their communities.	Mixed methods	*n* = 106 attendees including a mixed group of community members, staff of other health and community service organisations, and staff from Aboriginal Community-Controlled Health Organisations (ACCHOs)	We-Yarn is a suicide prevention gatekeeper training workshop—the workshop encouraged discussion and sharing of experiences, cultural tailoring of the program to include the seven domains of connection, and the use of the SCARF action plan (Suspect, Connect, Ask, Refer, Follow-Up).	We-Yarn appeared to be well-suited and culturally appropriate for Aboriginal suicide prevention gatekeeper training. There were significant improvements in participants’ self-reported knowledge and capacity to support someone struggling with social and emotional wellbeing problems. The workshops were valuable in strengthening knowledge and providing opportunities to discuss various approaches for culturally appropriate suicide prevention strategies.
Handley et al. [16] 2021	This paper describes the Good SPACE suicide prevention program and the lessons learned from delivering this program over a 14-year period (formerly known as the Farm-Link; see Perceval et al., 2020).	Commentary	Rural communities	Good SPACE is a suicide prevention program designed to prevent suicide through community and clinical education. The program educates rural community members to recognise the signs of suicide vulnerability and how to take action if they meet someone considering suicide.	A consistent finding was that approximately 80% of workshop attendees were females. The program was complemented by the development and co-design of variants to meet the needs of their intended audience.
Harris et al. [23] 2018	To display a selection of artworks in four small communities to engage and promote positive discussions about mental health.	Mixed methods survey	Visitor evaluation (*n* = 56)	The Rural Art Roadshow was a travelling program of artwork that visited rural communities to help improve community understanding of mental health, reduce stigma, and promote art-based mental health initiatives.	Each opening feature provided an opportunity for artists to speak about their experience of mental ill-health and creating their artwork. The art exhibition was well attended (*n* = 600 visitors), but with a low response rate to the evaluation surveys. There was a strong agreement that the art exhibition should be repeated annually.
Hearn et al. [19] 2016	To describe a community-developed Aboriginal model for early identification and referral of people with psychological distress and suicidal ideation.	Case study: descriptive	*n* = 12 support persons (No demographic data available)	The Jekkora model consists of recruitment and appointment of support persons, identifying people at risk, follow-up and referral by support persons, and expansion and sustainability. A series of training programs provided to support persons, such as: ASIST (Applied Suicide Intervention Skills Training), safeTALK, Living Hope Bereavement Support, and Support after Suicide.	The Jekkora model was developed by Aboriginal people for their community. It is a culturally acceptable, problem-specific, sustainable service for the early identification, treatment, and follow-up support for Aboriginal people in distress
Kennedy et al. [21] 2020	To evaluate the effectiveness of an intervention tailored for the farming community, designed to reduce stigma among male farm workers with a lived experience of suicide.	Mixed methods analysis	The Ripple Effect website had 12,755 visitors during the research period. *n* = 710 participants consented. Of these participants, *n* = 169 were from the target group (30–64-year-old males)	The Ripple Effect digital intervention was divided into five chapters, and email reminders were sent at designated time points to encourage completion. It included personal stories, videos, education, and personal goal setting.	The intervention was far-reaching. There were no identified changes in perceived stigma using quantitative measures; however, behavioural/ attitude changes emerged in the qualitative data. This program was successful in reaching their targeted population, along with other groups in rural Australia.
Perceval et al. [24] 2020	To evaluate a wellbeing and suicide prevention education workshop, SCARF (Suspect, Connect, Ask, Refer, Follow-Up), developed for Australian farming and rural communities.	Quantitative pre- and post-intervention evaluation	*n* = 255 (153 females, 102 males); mean age: 44.4 years	Wellbeing and suicide prevention education workshop—SCARF (Suspect, Connect, Ask, Refer, Follow-Up)—specifically developed for Australian farming and rural communities	There was a significant increase in suicide literacy and confidence immediately post-workshop, which remained at the 3-month follow-up. Mental wellbeing was also significantly improved at the 3-month follow-up. There were no changes to the overall score on the Stigma of Suicide Scale (SOSS).
Powell et al. [25] 2019	To examine the implementation of a community-driven mental health and wellbeing initiative in northern New South Wales, which began in response to a geographic cluster of local suicides.	Mixed methods	Review of 65 project documents (Quantitative data) Semi-structured interviews with 99 local stakeholders	“Our Healthy Clarence” is described as a novel, low-cost, small, bottom-up, locally driven approach. It was developed in response to a geographic cluster of local suicides. A stakeholder group formed to develop and enact the community mental health and wellbeing plan.	Stakeholders reported increased community agency, collaboration, optimism, and willingness to discuss mental health, suicide, and help-seeking. This initiative could serve as a model for other communities to address suicide and self-harm, and improve wellbeing.
Snodgrass et al. [20] 2020	To evaluate Deadly Thinking, which is a social and emotional wellbeing promotion program targeted at remote and rural Aboriginal and Torres Strait Islander communities.	Quantitative pre- and post-intervention evaluation	*n* = 413 participants across 40 locations in rural and remote Australia (*n* = 263 females, *n* = 114 males, *n* = 36 missing data)	Deadly Thinking aims to improve emotional health literacy, psychological wellbeing, and attitudes towards help-seeking associated with emotional ill-health.	Overall, there were low rates of marked distress in groups, and participants reported positive perceptions of community safety and wellbeing. Participants considered that that the workshop would help them to support others experiencing an emotional health crisis and improve their knowledge.

**Table 4:** Characteristics of suicide prevention initiatives.

Author and Year	Frequency of Program	Targeted Audience	Types of Activities	Evaluation Plan/Measurement/Tool	Program Strategies	Lessons Learnt
Barnett et al. [22] 2019	Art exhibition remained open for one week in each venue. Opening events were held in the evening and lasted 1–2 h.	Four small rural communities in Tasmania, Australia.	Art exhibition—22 pieces of art selected from the annual “Minds Do Matter” exhibition in Launceston.	Semi-structured interviews with the artists. Visitor survey of 6 statements on a Likert Scale and 3 open-ended questions.	The program promoted art as therapy and used the community event to foster social inclusion and have positive conversations about mental health. Entry to the exhibition was free.	Quality improvement suggestions included other mediums of disseminating the art, such as the use of social media or discussion boards. Future events should include a promotion and publicity plan tailored to each community.
Calabria et al. [17] 2020	The Community Reinforcement Approach (CRA) was offered to clients individually (60 min sessions) or in groups (90 min sessions). Six weekly sessions were planned, with the option of additional individual sessions.	Aboriginal and non- Aboriginal clients aged 18 years and over who attended a non- Aboriginal-focused community-based drug and alcohol treatment service in rural New South Wales.	Cognitive behavioural therapy (CBT) within a group setting or on an individual basis.	Outcome measures were collected at baseline, 4 weeks, 3 months, and 6 months. Kessler-5, Growth Empowerment Measure (GEM), and Alcohol, Smoking, and Substance Involvement Screening Test (ASSIST)	Therapists were local people who are known and trusted by the community. Aboriginal health workers were involved in the delivery of CRA to Aboriginal clients. CRA was embedded into routine practice.	This CBT approach also included training in skills that are transferable to other areas of life, such as communication, and may have contributed to improvements in psychological wellbeing, signifying the importance of building individual capacity.
Davies et al. [18] 2020	Six We-Yarn workshops that took approximately 6 h each over a period of 6 months.	Aboriginal people and those who work with Aboriginal communities and people in rural New South Wales.	Culturally safe suicide prevention skills training.	Self-rated responses on a 5-point Likert scale about their capacity and confidence to respond to a person at risk of suicide. Open-ended questions about the workshop. Focus groups.	Each workshop was facilitated by an experienced non-Aboriginal suicide prevention trainer and an experienced Aboriginal facilitator. Both facilitators had lived experience of suicide.	The sharing of the facilitators’ lived experiences was vital to instigating discussion and connecting with the workshop participants. Ongoing strategies should be community-led, and programs such as We-Yarn should be part of a multifaceted suicide prevention strategy.
Handley et al. [16] 2021	Good SPACE is a 4-h program.	Farmers, Aboriginal people, and general rural community members.	Education/ workshops	Evaluations included surveys/interviews following workshops.	The Good SPACE program was supplemented by other specialist training for GPs and clinicians to strengthen the local response.	Evaluation did not consider how to address the bigger issues of how to improve target audience reach, local engagement, and connection to services. General promotional approaches tended to recruit participants who were already well-versed in mental health.
Harris et al. [23] 2018	Art exhibition remained open for one week in each venue. Opening events were held in the evening and lasted 1–2 h.	Four small rural communities in Tasmania, Australia.	Art exhibition—22 pieces of art selected from the annual “Minds Do Matter” exhibition in Launceston.	Short survey of 6 statements on a Likert Scale and 3 open-ended questions.	The program promoted art as therapy and used the community event to foster social inclusion and have positive conversations about mental health. Entry to the exhibition was free.	Feedback indicated that the public wished to embed the exhibition in the local community, in partnership with local governments, businesses, schools, and health services. Suggestions from the public on how to improve future events included the need to have more interactive activities, the provision of further information on how to obtain mental health advice and services and increasing the reach of the roadshow.
Hearn et al. [19] 2016	Training programs lasted between 2 and 4 h, and were delivered to 12 individuals.	Posters and flyers were used to recruit interested people from the local community in Echuca, Victoria. After training, 10 individuals were selected to form the first group of voluntary trained support persons (VTSPs).	The VTSPs made weekly telephone calls to a referred person for 3 months using 5 questions to guide casual conversation. An Aboriginal health worker was notified if the person demonstrated sign of distress. Referrals were made by a GP.	Program evaluation plan not specified. VTSPs documented key responses and attended a monthly debriefing session.	At the end of the 3 months, community members who were supported were encouraged to take up the role of support persons for others in the community, and to undergo training.	By including people with a lived experience after their experience with the program, this model fosters community empowerment. Helps to establish social connectedness, wellbeing, and community resilience within Aboriginal communities.
Kennedy et al. [21] 2020	Online intervention estimated to take 2.5 to 4 h to complete; recommended to be completed in a few sessions over a couple of weeks.	The focus was on a male population (aged 30–64 years) in farming communities; however, participation in the intervention was expanded to include all adults (male and female).	Digital intervention consisting of five chapters.	Stigma of Suicide Scale; Literacy of Suicide Scale. Online feedback survey using both qualitative and quantitative questions.	Partners and stakeholders with links to the farming community were recruited to assist in sharing information about the Ripple Effect across rural networks, such as social media, local media, industry newsletters, community presentations, sporting clubs, and information flyers. A Community Champions Network was also developed to promote the project.	The previously identified evidence of association between increasing mental health literacy and decreasing mental health stigma may not apply to suicide literacy and suicide stigma. Support services should be easily accessible and should demonstrate an understanding of farming life and work when delivered within a rural context.
Perceval et al. [24] 2020	4-h workshop delivered free of charge.	Australian farming and rural communities in New South Wales.	Education.	Literacy of Suicide Scale, Stigma of Suicide Scale, Warwick–Edinburgh Mental Wellbeing Scale, confidence scale (developed for the study).	The program was delivered to frontline agricultural professionals, including agribusiness bankers, rural financial counsellors and accountants, those working with farming organisations—such as New South Wales (NSW) Farmers or Local Land Services—staff from employment, disability and care agencies, chaplains, and farming community groups.	Refresher training could be useful, as the mean scores for literacy and confidence dropped after 3 months despite a significant increase post-workshop. The SCARF program has since been updated to CARE (Connect, Ask, Refer, Encourage), signifying the importance of using contemporary evidence to inform suicide prevention programs.
Powell et al. [25] 2019	In early 2016, a steering group was formed to implement Our Healthy Clarence. This initiative has operated for two years and has five key objectives to address community mental health and wellbeing.	Members of the Clarence Valley Local Government area in New South Wales (~51,750 residents).	Community workshops. New services: a headspace centre; pop-up information and referral hubs in community centres. 2000 people received training in mental health literacy and suicide prevention in the community and workplaces. Standalone community events and connection with other community events and partners. Strength-based messaging through media. Sharing of Mindframe guidelines to media outlets.	Formative evaluation— use of 65 project documents and 36 semi-structured interviews with local stakeholders. No evaluation of the impact of the initiative.	The initiative was based on the principles of public health and community development. Factors that contributed to its success included leadership support, clarity of purpose, a paid independent coordinator, community involvement, and transparent governance.	To be sustainable, initiatives must respond to the local context and build on local assets if they are to be relevant and sustainable. Governance and structure were important to the success of the initiative. The multidimensional nature of the program denotes challenges and various complexities in evaluating the initiative, as statistics such as suicide rates or hospital admissions do not reflect the objectives of the initiative.
Snodgrass et al. [20] 2020	Two phases: group-based 1-day community workshop, or 2-day Train the Trainer (TTT) workshop	Aboriginal and Torres Strait Islander adults	Workshops	5 items adapted from Shaw and d’Abb’s community health scale, Kessler-5, MINI Suicidal Scale, a modified version of the Alcohol, Smoking, and Substance Involvement Screening Test, help-seeking intentions, and workshop feedback survey.	Deadly Thinking includes the opportunity for participants to discuss common sources of stress, such as family, employment, racism, discrimination, anxiety, depression, stigma, and suicide. It was developed in conjunction with local Aboriginal communities through a previous project.	There were a lower number of males who participated in the program; thus, the authors were unable to determine the extent to which the program was acceptable to men. It is recommended that providing a workshop specifically designed for men can encourage increased male attendance, creating a safe environment to discuss sensitive topics.

### 3.2. Key Characteristics of Suicide Prevention Initiatives

Workshops were the most frequent mode of intervention within the 10 studies. A four-hour workshop that featured in the works of both Handley et al. [16] and Perceval et al. [26] was delivered free of charge to participants, along with the six We-Yarn workshops over six months in the work of Davies et al. [18]. The workshops delivered by Snodgrass et al. [20] comprised either a group-based one-day community workshop or a two-day Train the Trainer (TTT) workshop. In the study of Calabria et al. [17], cognitive behavioural therapy (CBT) was offered over a six-week period, either in a group setting or on an individual basis. The design of this program was targeted towards adults requiring community-based drug and alcohol treatment services.

The promotion of art as a therapeutic intervention was described by Barnett et al. [22] and Harris et al. [23]. The art exhibition travelled to four small rural communities in Tasmania, featuring artworks inspired by artists who discussed their mental health journey with the community. The Ripple Effect digital intervention of Kennedy et al. [21] was designed to reduce suicide stigma in farming communities. Hearn et al. [19] described the Jekkora model, which focuses on training people to become voluntary trained support persons, to assist with identifying and referring people at risk of suicide. The “*Our Healthy Clarence*” initiative of Powell et al. [25] consisted of a range of activities such as community workshops, new services and referral hubs, mental health literacy training, and standalone community events. This complex initiative was developed in response to a geographic cluster of suicides in Clarence Valley.

### 3.3. Program Evaluation

The evaluation of these suicide prevention initiatives varied. Perceval et al. [24] and Kennedy et al. [21] both incorporated validated tools such as the Literacy of Suicide Scale and the Stigma of Suicide Scale as part of their evaluation process. Perceval et al. [24] also incorporated the Warwick–Edinburgh Mental Wellbeing Scale, along with a generalised confidence scale. Calabria et al. [17] incorporated substance-specific scales including the Alcohol, Smoking, and Substance Involvement Screening Test (ASSIST), as well as the Growth Empowerment Measure (GEM) and the Kessler-5, which is a measure of psychological distress.

A formative evaluation was completed by Powell et al. [25], using 65 project documents and 36 semi-structured interviews with local stakeholders. There was no evaluation of the impact of their suicide prevention initiative. An evaluation process of the program was not discussed in the work of Hearn et al. [19]; however, the authors discussed a monthly debriefing process for the support persons. Specific program feedback was sought in other studies using a range of Likert scales, focus groups, and open-ended questions [16,18,22,23].

### 3.4. Program Strategies

The suicide prevention initiatives used a range of strategies to engage their targeted audience.

Most activities that were intended for Aboriginal and Torres Strait Islander people involved the help of Aboriginal and Torres Strait Islander workers or people who were trusted by the community in facilitating and delivering the program [17,18,20]. In addition, the facilitators in the study of Davies et al. [18] also had the lived experience of suicide. Another strategy that was reported by Hearn et al. [19] was the encouragement of participants to take up the challenge of becoming a support person after the completion of the 3-month program. This encouraged program sustainability and increased the inclusion of facilitators with the lived experience of suicide and psychological distress. Additional specialist training was implemented for general practitioners and clinicians to strengthen the local community response in the work of Handley et al. [16], whereas in the work of Kennedy et al. [21] the use of a Community Champions Network was included to promote uptake of the digital intervention. Art was used as a creative outlet for artists to illustrate their mental health experiences, and to facilitate positive conversations about mental health, which promoted social inclusion in the rural community [22,23].

### 3.5. Key Findings of Initiatives

The qualitative findings of Barnett et al. [22] indicated that the *Rural Art Roadshow* demonstrated positive social and personal benefits for the participating artists. Similarly, attendance rates (*n* = 600) and visitor evaluations showed strong agreement that this art exhibition should be repeated annually [23]. Although there were no identified changes in perceived stigma using quantitative measures in the work of Kennedy et al. [21], qualitative data demonstrated behavioural and attitude changes among participants after the digital intervention.

The *Jekkora* model was described as a culturally acceptable, problem-specific, sustainable service for Aboriginal and Torres Strait Islander people in distress [19]. The paper in question discussed the *Jekkora* model; however, an evaluation of this program was not described. The Aboriginal-adapted Community Reinforcement Approach (CRA) in the work of Calabria et al. [17] was associated with statistically significant reductions in the use of alcohol, tobacco, cannabis, amphetamines, and over the counter medications, as well as levels of psychological distress.

Following the six We-Yarn workshops of Davies et al. [18], significant improvements were demonstrated in participants’ self-reported knowledge and capacity to support someone struggling with social and emotional wellbeing issues. Similarly, in the work of Perceval et al. [24], there was a significant increase in suicide literacy and confidence immediately post-workshop, which remained significant at the 3-month follow-up evaluation. Despite reported significant improvements to mental wellbeing at the 3-month follow-up, there were no demonstrated changes to the overall score on the Stigma of Suicide Scale [24]. In the work of Snodgrass et al. [20], there were low rates of marked distress in groups; however, participants considered that the workshop would help them to support others experiencing an emotional health crisis.

Stakeholders from the *Our Healthy Clarence* project reported increased community agency, collaboration, optimism, and willingness to discuss mental health, suicide, and help-seeking [25]. The authors reported that this initiative could serve as a model for other communities to address suicide and self-harm behaviours and improve wellbeing.

## 4. Discussion

The aim of this scoping review was to explore the literature regarding community-based suicide prevention initiatives for adults in Australian rural and regional communities. The authors intended to explore the key characteristics of these initiatives and how they are measured or evaluated. Given that a quality appraisal is usually not conducted in scoping reviews [12], a determination could not be made as to whether the findings of the included studies could be generalised to other settings. Overall, the findings from the suicide prevention initiatives were largely positive; however, the inclusion of only 10 articles in this review suggests a dearth of studies pertaining to this topic in an Australian context. This is despite the fact that there is anecdotal evidence of an increased number of Australian rural community co-produced mental health initiatives [10]. Stakeholders and facilitators of community-based suicide prevention programs should be encouraged to use a formal evaluation process and to publish their research findings. The evaluation of the suicide prevention initiatives in this review varied, depending on the aims of the program. Complex initiatives such as that of Powell et al. [25] reported difficulty in evaluating their initiatives due to the multidimensional nature of the program. It is imperative that suicide prevention programs include adequate evaluation strategies for continuous quality improvement, resulting in improved health outcomes for consumers [8].

The facilitators’ lived experience of suicide was noted as a positive inclusion by Davies et al. [18]. The authors described how the sharing of the lived experience of the facilitators was vital to instigating workshop discussions and connecting with participants. Jones et al. [27] noted that although including people with a lived experience of suicide can be a powerful learning experience, it should be done in an appropriate and safe manner. Similarly, most of the suicide prevention programs that were intended for Aboriginal and Torres Strait Islander people used an Aboriginal and Torres Strait Islander facilitator, and the authors described cultural adaptation of the program. In 2018, the suicide rates of Aboriginal and Torres Strait Islander people were almost double that of non-indigenous Australians [28], illustrating the importance of providing culturally appropriate activities within rural communities. Previous studies recommended that suicide prevention programs for Aboriginal and Torres Strait Islander people should have a holistic approach and consider the social, emotional, and spiritual elements of community wellbeing [29]. Although the *Jekkora* model is in its early stages of implementation, it has the potential to increase community resilience and social connectedness within Aboriginal and Torres Strait Islander communities [19].

The suicide prevention initiatives in this review described favourable outcomes, yet there were concerns that some programs did not reach their target audience. In the work of Handley et al. [16], a consistent finding was that approximately 80% of workshop attendees were females. Statistics show that males are three times as likely to die by suicide compared to females [4]. Kennedy et al. [21] reported that their digital intervention was successful in reaching their target audience (i.e., male farmers in rural communities). This indicates that further consideration needs to be given to tailoring the type of activity to the targeted population. For example, rural males may not participate in a workshop on suicide prevention, but they may attend other activities of interest, such as an informal barbecue [16]. Perceval et al. [24] and Kennedy et al. [21] described their key strategies for engaging their target audience, including recruiting people who were specifically working in primary production, or as bankers or accountants. Kennedy et al. [21] also discussed the use of social media, flyers, presentations at sporting clubs, and the use of a Community Champion to promote their digital intervention. This is a positive example of using rural community resources to reach the intended population.

Future recommendations for some of the initiatives included community interactions to ensure long-term sustainability. Powell et al. [25] discussed how initiatives must respond to the local context and build on local assets if they are to be sustainable. This was also discussed by Harris et al. [23], where program feedback included partnering with local governments, businesses, schools, and health services to improve uptake of the art exhibition. Additional recommendations by Perceval et al. [24] included the need for refresher training, given that quantitative measures showed a decrease in scores in the 3 months post-intervention. Other Australian studies noted that there was little evidence of learning from preceding rural community co-produced mental health initiatives [10]. This is of concern, considering the high number of deaths by suicide in rural and regional Australian communities. There are complex social, cultural, and ethical attributes underlying psychological distress in rural communities that are not supported by the mental health system [30]. Future community-based suicide prevention initiatives in rural communities should have a holistic approach that is tailored to the community’s needs. As previously discussed, program organisers of suicide prevention initiatives should be encouraged to formally evaluate and publish their findings to provide opportunities for others to learn from their experiences.

## 5. Limitations

A limitation to this study is that only studies from an Australian context were included; therefore, these results may not be generalisable to an international perspective. It is possible that some national suicide prevention initiatives may have provided further insights; however, studies that were not rural- or regional-based were excluded from this review. Scoping reviews are not intended to be a definitive synthesis of the literature but can be useful in identifying research gaps in the literature [31,32]. This research builds upon previous reviews by exploring generalised community-based suicide prevention programs in rural and regional Australia and providing recommendations for the delivery of future programs.

## 6. Conclusions

Suicide rates are higher in rural and regional areas compared to their metropolitan counterparts, exacerbated by difficulties in accessing treatment and support services. Community-based suicide prevention initiatives in these areas require a holistic approach tailored to the local community’s needs. This is essential for long-term program sustainability and to cater to the program’s target audience, e.g., rural adult males. Furthermore, continued evaluation of community-based rural and regional suicide prevention initiatives is imperative to promote continued improvement of the quality of these programs. This scoping review highlights the dearth of literature surrounding community-based suicide prevention initiatives in rural and regional Australia. Further research and continued evaluation of the efficacy of suicide prevention programs are recommended.

## Figures and Tables

**Table 1:** Search terms.

Search Term	Variations
Suicid *	Suicide prevention Suicide reduction Suicide intervention Mental health Mental illness Mental disorder
Adult *	Older Middle aged Farm *
Initiative *	Program * Intervention * Strateg *
Regional	Rural Remote Community Country
NOT child *	Paediatric Pediatric Young Adolesc * Teenager

Key: * = truncated search term.

**Table 2:** Inclusion and exclusion criteria.

**Inclusion Criteria**	**Exclusion Criteria**
Peer-reviewed publications Studies published between 2012 and 2022 English language Full-text articles Studies conducted in Australia Adults aged ≥18 years Rural/regional areas only Community-based	Metropolitan areas Hospital-based suicide prevention initiatives (inpatient/outpatient) Adolescent or youth population (<18 years) Studies focused on psychometric or measurement tools

## Data Availability

Data sharing is not applicable as no new data was created for this study.
